# Independent and Opposite Associations Between Branched-Chain Amino Acids and Lysophosphatidylcholines With Incident Diabetes in Thais

**DOI:** 10.3390/metabo10020076

**Published:** 2020-02-20

**Authors:** La-or Chailurkit, Nitchawat Paiyabhroma, Piyamit Sritara, Prin Vathesatogkit, Sukit Yamwong, Nisakron Thonmung, Boonsong Ongphiphadhanakul

**Affiliations:** 1 Department of Medicine, Faculty of Medicine, Ramathibodi Hospital, Mahidol University, Rama 6th Road, Bangkok 10400, Thailandnitchawatp57@nu.ac.th (N.P.); piyamit.sri@mahidol.ac.th (P.S.); prin.vat@mahidol.ac.th (P.V.); sukit.yam@mahidol.ac.th (S.Y.); 2 Office of Research, Academic Affairs and Innovations, Faculty of Medicine, Ramathibodi Hospital, Mahidol University, Bangkok 10400, Thailand; nisakorn.tho@mahidol.ac.th

**Keywords:** branched-chain amino acids, lysophosphatidylcholine, diabetes

## Abstract

Branched-chain amino acids (BCAAs) and lysophosphatidylcholines (LPCs) have been reported to be associated with diabetes. The purpose of the present study was to investigate the relative contributions of BCAAs and LPCs to the progression of prediabetes to diabetes using a targeted metabolomic approach. This study was part of a health survey of employees of the Electricity Generating Authority of Thailand (*n* = 79; nine females and 70 males). A targeted metabolomics analysis was performed using an AbsoluteIDQ^®^ p180 kit, flow injection analysis, and liquid chromatography-tandem mass spectrometry. The highest variable importance in projection (VIP) scores for the progression to diabetes of the amino acids and phospholipids were associated with isoleucine and LPC acyl C28:1, respectively. Using logistic regression analysis, we found that high baseline isoleucine concentration was associated with a higher incidence of diabetes, while high LPC acyl 28:1 was associated with a lower incidence. Isoleucine and LPC acyl 28:1 were independently associated with incident diabetes in a model that also included conventional risk factors for diabetes (baseline fasting plasma glucose (FPG), age, sex, and body mass index (BMI)). In addition, isoleucine and LPC acyl 28:1 were independently associated with serum HbA1c 5 years later in a robust regression model that also included baseline FPG, age, sex, and BMI. Isoleucine, LPC acyl 28:1, age, and FPG were significantly associated with HbA1c at this time. In conclusion, these results provide evidence that isoleucine and LPC acyl C28:1 have respective positive and negative independent associations with incident diabetes.

## 1. Introduction

Previous studies have identified associations between branched-chain amino acids (BCAAs) and type 2 diabetes. The biologic pathways that may mediate the potential involvement of BCAAs in the pathogenesis of type 2 diabetes are unclear but are likely to involve BCAA-mediated increase in insulin resistance [1]. Many mechanisms have been proposed for BCAA-medicated insulin resistance, but none have been proven to date. Some of the suggested mechanisms include activation of the mTORC1 complex, which is sensitive to metabolic clues, including amino acids [2], increase in insulin secretion and compensatory insulin resistance [3], and competition for fatty acid oxidation [4]. Most studies on the role of BCAAs in diabetes have been performed in Caucasians. In Asian populations, where lifestyle and dietary factors may differ, a number of studies have also shown the association between BCAAs and diabetes. For examples, a recent study conducted in China demonstrated similar associations of BCAAs and aromatic amino acids (AAAs) with diabetes in the local population [5]. Dietary intake of BCAAs and AAAs (tyrosine in particular) has been demonstrated to be related to the risk of diabetes in Japanese subjects, which suggests the causal role of BCAAs and AAAs in the pathogenesis of type 2 diabetes [6]. In a cross-sectional study in Singapore, it was found that insulin resistance was correlated with increased levels of amino acids, particularly BCAAs [7].

In addition to BCAAs, the composition of the lipidome has been shown to differ according to glycemic status [8], and phospholipids such as lysophosphatidylcholines (LPCs) have been demonstrated to be associated with obesity and diabetes [9]. In a study in the Chinese population, lipids, including triacylglycerols, lyso-phosphatidylinositols, phosphatidylcholines, polyunsaturated fatty acid–plasmalogen phosphatidylethanolamines, and cholesteryl esters, were shown to be related to the future development of type 2 diabetes [10]. Moreover, it has been suggested that LPCs may mediate the metabolic effects of metformin [11,12].

Furthermore, in rodent models, BCAAs interact with lipids to enhance insulin resistance, while BCAAs alone have little effect [13]. However, in humans, the relative effect of BCAAs and LPCs in the progression of prediabetes to diabetes is unclear at present. Therefore, we aimed to use a targeted metabolomic approach to determine the relative contributions of BCAAs and LPCs to the progression of prediabetes to diabetes in Thai people.

## 2. Materials and Methods

### 2.1. Subjects

Participants were recruited from the population studied in the Electricity Generating Authority of Thailand (EGAT) study in 2009 (EGAT 3). Details of the study cohort have been published previously [14]. Briefly, the subjects in the cohort were employees of EGAT who volunteered to participate in a health survey. All participants completed a medical evaluation and had routine laboratory investigations, including urinalysis. Blood was drawn after a 12 h fast. In summary, there were 3 EGAT cohorts in total. In 1985, 3499 EGAT workers (half of the total employees) were randomly enrolled as EGAT 1 cohort. In 1998, 2999 employees were randomly enrolled as EGAT 2 cohort. In 2009, 2584 participants were recruited to the EGAT 3 cohort. EGAT 3 was resurveyed in 2014. Each time, the same individuals were contacted by telephone and invitation letter to attend the follow-up examination, or else information about the cause of death was sought for those known to have died during the interim period. At each follow-up visit, the subjects underwent similar medical evaluations and had the same routine laboratory investigations as the baseline visit.

A total of 79 participants (9 females and 70 males) aged 27–54 years were enrolled in this retrospective study. The participants were randomly selected from those who did not have a previous history of diabetes and had a fasting plasma glucose (FPG) ≥ the 85th percentile (5.4 mmol/L) but < 7 mmol/L. The body mass and height of each participant were measured using standard techniques. Body mass index (BMI) was calculated using body mass (kg)/height^2^ (m). Fasting blood samples were obtained and kept at −80 °C until analysis. The progression to diabetes over the following 5 years was assessed by the measurement of glycated hemoglobin (HbA1c; diagnostic threshold 6.5%).

### 2.2. Ethical Approval

The Committee on Human Rights Related to Research Involving Human Subjects, Faculty of Medicine, Ramathibodi Hospital, Mahidol University approved the study, and it conformed with the provisions of the Declaration of Helsinki (as revised in Fortaleza, Brazil, October 2013). The ethical approval code is MURA2019/290 and the date of approval is 4 April 2019. All participants gave their written informed consent before participating in the study.

### 2.3. Biochemical Measurements

FPG was analyzed using an automated random-access chemistry analyzer (Architect c8000, Abbott Laboratories, Abbott Park, IL, USA). HbA1c was assayed using a turbidimetric inhibition immunoassay (Tina-quant Hemoglobin A1c Gen.3 kit, Mannheim, Germany) on a Cobas c502 module (Roche Diagnostics GmbH, Mannheim, Germany).

### 2.4. Targeted Metabolite Assessment

Targeted metabolomics of serum samples was undertaken using an AbsoluteIDQ^®^ p180 kit (Biocrates Life Sciences AG, Innsbruck, Austria), involving separation by liquid chromatography (LC), followed by tandem mass spectrometry (MS/MS) measurements. Sample preparation was performed in accordance with the manufacturers’ instructions and the samples were analyzed on an QTRAP 5500 mass spectrometer (ABsciex, Framingham, MA, USA), using electrospray ionization and operating in the multiple reaction monitoring (MRM) mode, coupled to an Agilent HPLC 1260 Series (Agilent Technologies, Santa Clara, CA, USA) equipped with an Agilent Zobax Eclipse XDB C18, 3.0 mm × 100 mm, 3.5 μm column, and a Phenomenex C18, 4.0 mm × 3.0 mm SecurityGuard column. Amino acids and biogenic amines were analyzed by LC-MS/MS in positive mode and other metabolites were analyzed using flow injection analysis coupled to tandem mass spectrometry (FIA-MS/MS) in both positive and negative modes. Metabolites were quantified according to the manufacturer’s protocol, using the MetIDQ™ Carbon software for targeted metabolomic data processing and management. This assay quantifies up to 188 targeted metabolites that encompass the following compound classes: 21 amino acids, 21 biogenic amines, 40 acylcarnitines, 90 glycerophospholipids, 15 sphingolipids, and one hexose. Lipid side-chain composition is given in the format Cx:y, where x denotes the number of carbons in the side chain and y the number of double bonds.

### 2.5. Statistical Analysis

Statistical analysis was performed and predictive models were constructed using Rstudio version 1.0.136 and R version 3.3.2 (RStudio Inc., Boston, MA, USA). Comparisons between groups were measured by the Student’s t test. Feature selection was performed using a partial least squares-based algorithm for parsimonious variable selection [15], with the variable importance in projection (VIP) threshold set to 1.5. Partial least squares discriminant analysis (PLS-DA) modeling of baseline metabolites for the presence or absence of diabetes at 5 years, which was performed with the ropls R package. The significance of R2 and Q2 values was estimated by permutation testing. The features selected were then analyzed for statistical significance using univariate logistic regression or linear regression analyses, as appropriate. *p* < 0.05 was considered statistically significant.

## 3. Results

The clinical characteristics of the participants are shown in Table 1. Their mean age was 43.7 ± 6.8 years, and most (88.6%) were males, due to the demographic structure of the workforce in the organization where the participants were recruited. The participants were generally obese, having a mean (±SD) BMI of 27.1 ± 4.4 kg/m^2^. Their mean baseline FPG was 107.5 ± 7.9 mg/dL. Thirty-seven (47.4%) had developed frank diabetes after 5 years and the mean HbA1c of the study population at this time point was 6.7 ± 1.7%.

**Figure 1:**
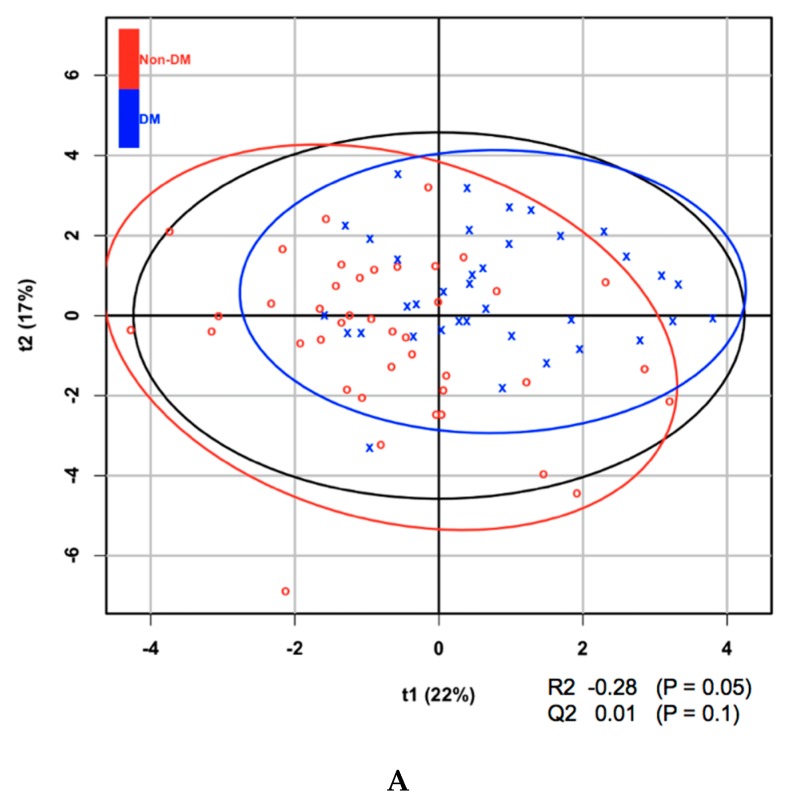

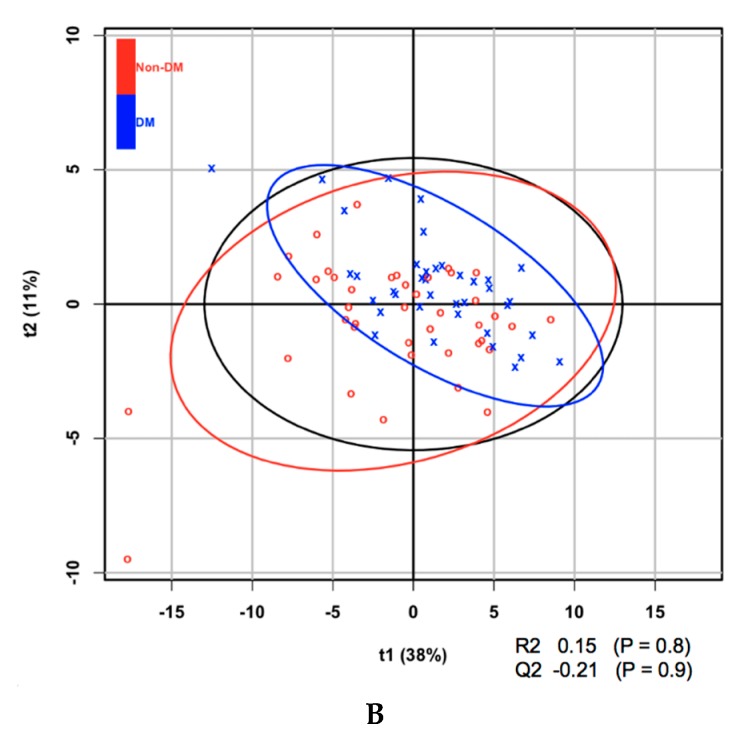
(**A**) Partial least squares discriminant analysis (PLS-DA) score plot of amino acids at baseline for the development of diabetes within 5 years. (**B**) PLS-DA score plot of lysophosphatidylcholines (LPCs) at baseline for the development of diabetes within 5 years.

Metabolites at baseline were analyzed using partial least squares discriminant analysis (PLS-DA) to classify subjects according to the presence or absence of diabetes after 5 years and ranked according to their VIP score. Of the 21 amino acids, those that contributed to the progression to diabetes, on the basis of their VIP score > 1.5, were isoleucine, leucine, and valine (Table 2). It is of note that all amino acids with a VIP score > 1.5 are BCAAs. BCAAs have been demonstrated in a number of studies to be associated with the prevalence, as well as incidence, of diabetes. Aliphatic amino acids, which have also been shown to be associated with diabetes in some previous studies, were not found to have sufficiently high VIP scores in the present study. In addition, it was found that one of 14 LPCs (LPC acyl C28:1, which had the highest VIP score) and a number of phosphatidylcholines had VIP scores surpassing the 1.5 threshold. The score plot and associated R2, Q2, and *P*-values from the permutation test are shown in Figure 1.

Table 3 shows the difference in clinical characteristics as well as baseline BCAAs and LPCs whose VIP scores were > 1.5 according to diabetes status within 5 years. Among the clinical characteristics, BMI and FPG significantly differed between groups. All BCAAs with VIP scores > 1.5 were significantly different between the 2 groups, while there were variations in the statistical significance of LPCs with VIP scores > 1.5.

Table 4 shows the Pearson’s correlation matrix for BCAAs, LPC acyl C28:1, sex, and baseline age and BMI. It is noteworthy that all three BCAAs were correlated, whereas there was no correlation between LPC acyl C28:1 and the BCAAs. Furthermore, LPC C28:1 was not correlated with age, sex, or BMI.

Using logistic regression analysis to determine the independent influence of BCAAs, represented by isoleucine, which had the highest VIP score among the BCAAs and LPC acyl C28:1, it was found that baseline isoleucine was associated with a higher incidence of diabetes, whereas LPC acyl C28:1 was associated with a lower incidence of diabetes after 5 years (Table 5). Isoleucine and LPC acyl C28:1 were independently associated with incident diabetes in a model that included the conventional risk factors for diabetes (baseline FPG, age, sex, and BMI; Table 4). In addition to isoleucine and LPC acyl C28:1, baseline FPG was the only other variable that was significantly associated with incident diabetes. In addition, isoleucine and LPC acyl C28:1 were independently associated with HbA1c after 5 years in a robust regression model that included baseline FPG, age, sex, and BMI (Table 6). In this model, in addition to isoleucine and LPC acyl C28:1, age and FPG were significantly associated with HbA1c 5 years later, and BMI was close to statistical significance.

## 4. Discussion

Higher circulating BCAAs concentrations are associated with greater insulin resistance and type 2 diabetes [16,17]. With regard to Asian populations, however, a high BCAAs intake was found to reduce the risk of diabetes in a Japanese study [6], whereas higher BCAAs in a Chinese study [2] was related to higher risk of diabetes. Heterogeneity in dietary patterns across the world, an important determinants of population health [18], may be partly responsible for this discrepant finding. Nevertheless, the causal roles of BCAAs in this regard are supported by a number of intervention studies. For examples, BCAA restriction in Zucker fatty rats improves muscle insulin sensitivity [19]. Moreover, a reduced BCAA diet promotes rapid fat mass loss, as well as restores glucose tolerance and insulin sensitivity without calorie restriction in obese mice [20]. There are a number of mechanisms underlying the elevated circulating BCAAs and their effects on insulin resistance and type 2 diabetes. For examples, leucine has been demonstrated to lower skeletal adenosine monophosphate (AMP)-activated protein kinase activity [21]. Similarly, leucine deprivation improves hepatic insulin sensitivity by activating AMP-activated protein kinase [22]. Moreover, 3-hydroxyisobutyrate, a catabolic intermediate of the BCAA valine, enhances vascular fatty acid transport and causes insulin resistance [23]. In keeping with the findings of most previous studies, we have demonstrated an association between BCAAs and subsequent type 2 diabetes in Thai people.

LPCs are associated with lower cardiovascular mortality [24] and plasma LPC concentrations are lower in people with obesity and type 2 diabetes [4]. Our study adds to these previous findings by showing that the concentration of a specific LPC is associated with subsequent diabetes. Although the precise role of LPCs is uncertain, the identified association between this LPC and type 2 diabetes may imply that it has a causal role.

Lysophospholipids have been intensively investigated recently and found to possess hormone-like activity because they regulate a number of biologic processes [25]. Of all the lysophospholipids, LPCs are the most abundant in plasma and tissues. Several types of circulating LPC molecules with various acyl chains have been found in humans [26]. With regard to diabetes, LPC 18:1 has been shown to be a novel ligand of GPR119, regulating pancreatic insulin secretion [27,28]. In the present study, LPC 18:1 was not identified as an important predictor of incident diabetes, but LPC 28:1 was. Nevertheless, in terms of their cardiovascular effects, LPC 18:1, LPC 28:1, and other species have been found to be associated with better microvascular function after lipoprotein apheresis in lipidemic patients [29].

Although BCAAs and LPCs have previously been separately demonstrated to be associated with diabetes, their joint association has not been explored. In the present study, we found that isoleucine and LPC 28:1 were independent predictors of incident diabetes. Furthermore, the concentrations of all the BCAAs (leucine, isoleucine, and valine) highly correlate, and their concentrations also correlate with well-established risk factors for diabetes, such as BMI, complicating the analysis of their roles using multiple regression because of multicollinearity. This undermines the statistical significance of an independent variable, making a meaningful interpretation of the output difficult [30]. In contrast, LPC did not correlate with BCAAs or other covariates of diabetes, potentially making a causal role of LPC more likely. The negative association between LPC and incident diabetes remained significant after adjusting for BCAAs concentrations and common covariates of type 2 diabetes.

There are a number of limitations in our study. The sample size is small and volunteers were recruited from a single location. Moreover, there was an imbalance in gender distribution due to the characteristics of the EGAT workforce. The generalizability of our findings is, therefore, limited. 

In conclusion, the present findings provide evidence that isoleucine and LPC acyl C28:1 are independently associated with the risk of diabetes but in opposite directions.

## Figures and Tables

**Table 1:** Clinical characteristics of the study population (*n* = 79).

Variables	Mean ± Standard Deviation or Number (%)
Age (year)	43.7 ± 6.8
Male (%)	70 (88.6%)
Body mass index (kg/m^2^)	27.1 ± 0 4.4
Fasting plasma glucose (mmol/L)	5.97 ± 0.44

**Table 2:** Amino acids and phospholipids that had a variable importance in projection (VIP) score > 1.5 at baseline for the prediction of incident diabetes within 5 years.

Metabolite	VIP Score
**Amino acid**	
Isoleucine	2.1
Leucine	1.8
Valine	1.6
**Glycerophospholipid**	
Lysophosphatidylcholine acyl C28:1	1.92
Phosphatidylcholine diacyl C28:1	1.87
Phosphatidylcholine acyl-alkyl C40:1	1.83
Phosphatidylcholine diacyl C42:4	1.77
Phosphatidylcholine acyl-alkyl C38:2	1.77
Phosphatidylcholine acyl-alkyl C42:5	1.74
Phosphatidylcholine acyl-alkyl C40:5	1.73
Phosphatidylcholine acyl-alkyl C42:4	1.62
Phosphatidylcholine acyl-alkyl C36:1	1.61
Phosphatidylcholine diacyl C40:3	1.58
Phosphatidylcholine acyl-alkyl C40:3	1.55
Phosphatidylcholine acyl-alkyl C38:0	1.53
Phosphatidylcholine acyl-alkyl C40:2	1.51

In the format Cx:y, x denotes the number of carbons in the side chain and y the number of double bonds.

**Table 3:** The difference in clinical characteristics as well as baseline branched-chain amino acids (BCAAs) and LPCs whose VIP scores were > 1.5 according to diabetes (DM) status within 5 years. Note: BMI, body mass index; FPG, fasting plasma glucose.

Variables	Non-DM (*n* = 41)		DM (*n* = 38)		*p*-Value
	Mean	SE	Mean	SE	
Age (year)	43.10	1.11	43.89	1.05	0.60
Male (%)	86.10		88.90		0.68
BMI (kg/m^2^)	25.88	0.62	28.11	0.77	<0.05
FPG (mmol/L)	5.75	0.07	6.13	0.08	<0.001
Ile (nmol/L)	116.34	2.98	131.50	3.04	<0.01
Leu (nmol/L)	264.89	9.18	298.39	9.01	<0.05
Val (nmol/L)	320.00	5.67	339.50	5.46	<0.05
Lysophosphatidylcholine acyl C28:1 (nmol/L)	0.27	0.01	0.23	0.01	<0.05
Phosphatidylcholine diacyl C28:1 (nmol/L)	2.33	0.09	2.06	0.08	<0.05
Phosphatidylcholine acyl-alkyl C40:1 (nmol/L)	0.40	0.01	0.38	0.01	0.29
Phosphatidylcholine diacyl C42:4 (nmol/L)	0.68	0.03	0.59	0.02	<0.05
Phosphatidylcholine acyl-alkyl C38:1 (nmol/L)	2.73	0.29	2.82	0.28	0.82
Phosphatidylcholine acyl-alkyl C42:5 (nmol/L)	0.28	0.02	0.26	0.01	0.41
Phosphatidylcholine acyl-alkyl C40:5 (nmol/L)	9.15	0.52	8.49	0.45	0.34
Phosphatidylcholine acyl-alkyl C42:4 (nmol/L)	0.22	0.02	0.18	0.01	<0.05
Phosphatidylcholine acyl-alkyl C36:1 (nmol/L)	39.89	1.55	38.68	1.52	0.58
Phosphatidylcholine diacyl C40:3 (nmol/L)	1.36	0.20	0.94	0.06	0.06
Phosphatidylcholine acyl-alkyl C40:3 (nmol/L)	0.47	0.02	0.41	0.02	0.05
Phosphatidylcholine acyl-alkyl C38:0 (nmol/L)	2.80	0.11	2.70	0.12	0.53
Phosphatidylcholine acyl-alkyl C40:2 (nmol/L)	0.40	0.01	0.38	0.01	0.29

**Table 4:** Pearson’s correlation matrix for branched-chain amino acids (BCAAs), lysophosphatidylcholine (LPC) acyl C28:1, sex, and baseline age and body mass index (BMI).

Variable	Leucine	Isoleucine	Valine	LPC C28:1	Age	Sex	BMI
**Leucine**	1						
**Isoleucine**	0.87 *p* < 0.001	1					
**Valine**	0.68 *p* < 0.001	0.69 *p* < 0.01	1				
**LPC C28:1**	0.06 *p* = 0.59	0.05 *p* = 0.6	0.02 *p* = 0.88	1			
**Age**	−0.27 *p* < 0.05	−0.21 *p* = 0.06	−0.19 *p* = 0.09	0.08 *p* = 0.46	1		
**Sex**	−0.13 *p* = 0.23	−0.15 *p* = 0.18	−0.08 *p* = 0.46	−0.15 *p* = 0.18	−0.002 *p* = 0.99	1	
**BMI**	0.30 *p* < 0.01	0.31 *p* < 0.05	0.30 *p* < 0.01	−0.15 *p* = 0.17	−0.16 *p* = 0.14	0.24 *p* < 0.05	1

In the format Cx:y, x denotes the number of carbons in the side chain and y the number of double bonds. BCAA concentrations are highly correlated with the other parameters, with the exception of LPC acyl C28:1.

**Table 5:** Logistic regression model showing the independent influence of branched-chain amino acids (BCAA), represented by isoleucine, which had the highest variable importance in projection score among the BCAAs, and lysophosphatidylcholine (LPC) acyl C28:1 on the incidence of diabetes after 5 years.

Variables	Odds Ratio	95% Confidence Interval	*p*-Value
Isoleucine	1.00025	1.000003–1.00051	<0.05
LPC acyl C28:1	0.976	0.972–0.999	<0.05
Baseline FPG	1.080	1.009–1.057	<0.05
Baseline age	1.567	0.965–1.157	0.24
Baseline BMI	1.082	0.938–1.249	0.28
Female	0.936	0.140–6.256	0.95

Note: FPG, fasting plasma glucose; BMI, body mass index. In the format Cx:y, x denotes the number of carbons in the side chain and y the number of double bonds.

**Table 6:** Robust regression model showing the independent influence of isoleucine and lysophosphatidylcholine (LPC) acyl C28:1 on hemoglobin A1c 5 years later.

Variable	Coefficient (×10^−3^)	*p*-Value
Isoleucine	0.095	<0.05
LPC acyl C28:1	−5.15	<0.05
Baseline FPG	31.91	<0.05
Baseline age	32.81	<0.05
Baseline BMI	57.40	0.052
Female	−346.20	0.25

FPG, fasting plasma glucose; BMI, body mass index. In the format Cx:y, x denotes the number of carbons in the side chain and y the number of double bonds.
